# Lacrimispora brassicae sp. nov. isolated from fermented cabbage, and proposal of Clostridium indicum Gundawar et al. 2019 and Clostridium methoxybenzovorans Mechichi et al. 1999 as heterotypic synonyms of Lacrimispora amygdalina (Parshina et al. 2003) Haas and Blanchard 2020 and Lacrimispora indolis (McClung and McCoy 1957) Haas and Blanchard 2020, respectively

**DOI:** 10.1099/ijsem.0.006456

**Published:** 2024-07-17

**Authors:** Hisami Kobayashi, Yasuhiro Tanizawa, Mitsuo Sakamoto, Moriya Ohkuma, Masanori Tohno

**Affiliations:** 1 Institute of Livestock and Grassland Science, National Agriculture and Food Research Organization, Nasushiobara, Tochigi 329-2793, Japan; 2 Department of Informatics, National Institute of Genetics, Mishima, Shizuoka 411-8540, Japan; 3 Microbe Division/Japan Collection of Microorganisms, RIKEN BioResource Research Center, Tsukuba, Ibaraki 305-0074, Japan; 4 Research Center of Genetic Resources, National Agriculture and Food Research Organization, Tsukuba, Ibaraki 305-8602, Japan; 5 Innovative Animal Production System, University of Tsukuba, 305-8571 Tsukuba, Japan

**Keywords:** Bacillota, fermented cabbage, *Lacrimispora brassicae* sp. nov.

## Abstract

A Gram-stain-negative, endospore-forming, rod-shaped, indole-producing bacterial strain, designated YZC6^T^, was isolated from fermented cabbage. Strain YZC6^T^ grew at 10–37  °C, pH 5.5–8.5, and with up to 2  % (w/v) NaCl. The major cellular fatty acids were C_16 : 0_ and C_18 : 1_*cis* 11 dimethyl acetal. Phylogenetic analysis of the 16S rRNA gene revealed that strain YZC6^T^ belonged to the genus *Lacrimispora* and was closely related to *Lacrimispora aerotolerans* DSM 5434^T^ (98.3  % sequence similarity), *Lacrimispora saccharolytica* WM1^T^ (98.1  %), and *Lacrimispora algidixylanolytica* SPL73^T^ (98.1  %). The average nucleotide identity based on blast (below 87.8  %) and digital DNA–DNA hybridization (below 36.1 %) values between the novel isolate and its corresponding relatives showed that strain YZC6^T^ could be readily distinguished from its closely related species. Based on genotypic, phenotypic, and chemotaxonomic data, a novel *Lacrimispora* species, *Lacrimispora brassicae* sp. nov., was proposed, with YZC6^T^ as the type strain (=MAFF 212518^T^=JCM 32810^T^=DSM 112100^T^). This study also proposed *Clostridium indicum* Gundawar *et al*. 2019 as a later heterotypic synonym of *Lacrimispora amygdalina* (Parshina *et al*. 2003) Haas and Blanchard 2020 and *Clostridium methoxybenzovorans* Mechichi *et al*. 1999 as a later heterotypic synonym of *Lacrimispora indolis* (McClung and McCpy 1957) Haas and Blanchard 2020.

## Introduction

The genus *Lacrimispora* was reclassified based on phylogenetic and phylogenomic analyses and was transferred from the *Clostridium* XIVa cluster in 2020 [1]. Eight species of the genus *Lacrimispora* are currently recognized: *Lacrimispora sphenoides* (type species of this genus), *Lacrimispora aerotolerans*, *Lacrimispora algidixylanolytica*, *Lacrimispora amygdalina*, *Lacrimispora celerecrescens*, *Lacrimispora indolis*, *Lacrimispora saccharolytica*, and *Lacrimispora xylanolytica* (https://lpsn.dsmz.de). Many members of this genus are commonly Gram-stain-negative and spore-forming anaerobes [1].

Species of the genus *Lacrimispora* have attracted attention owing to their potential impact on human and animal health. For instance, *L. celerecrescens* has been linked to human infection [2,3], while *L. indolis* has been found to be enriched in the gut of dogs with diabetes mellitus [4].

*Lacrimispora* species have been isolated from various environmental sources, including human and animal clinical specimens, soil, human and animal faeces, sheep rumen, vacuum-packed raw lamb, anaerobic digester sludge, marine sediment, and sewage sludge [5]. In addition, they have been recovered from various plant samples [5,6]. In vacuum-packaged peeled potatoes, *Lacrimispora* was positively correlated with ethanol content, which is the most abundant spoilage marker [6].

The primary aim of the present study was to investigate the phenotypic characteristics of the novel strain YZC6^T^, isolated from fermented cabbage, and to determine the taxonomic relationship between the novel strain and its closely related species within the genus *Lacrimispora*. In this study, we determined that the reclassification of *Clostridium indicum* and *Clostridium methoxybenzovorans* was necessary. Another aim was to re-evaluate the taxonomic status of these species using phylogenetic analysis based on whole-genome-based metrics, such as average nucleotide identity (ANI), digital DNA–DNA hybridization (dDDH), and average amino acid identity (AAI).

## Methods

### Isolation and ecology

A sample of cabbage residue was collected from a local vegetable processing place in Yamato, Kanagawa, Japan (longitude 139° 27′ 50″, latitude 35° 29′ 55″) in 2016. The sample was stored in vacuum-sealed bags at room temperature in Nasushiobara, Tochigi, Japan (longitude 139° 55′ 49″, latitude 36° 55′ 9″) for 3 days. The stored cabbage residue was then transferred to sterile homogenization bags, and homogenates were prepared according to a previously described method [7]. The novel strain YZC6^T^ was isolated from the homogenates after incubation on a Clostridia count agar (Nissui Pharmaceuticals) for 3 days at 30  °C under anaerobic conditions (Anaero-Pack, Mitsubishi Gas Chemical). A pure isolate, designated as the type strain, was then examined using a reinforced clostridial medium (RCM; BD Difco) agar plate at 30  °C under anaerobic conditions (Anaero-Pack) and a peptone–yeast extract–glucose (PYG) medium [8] under an H_2_/CO_2_/N_2_ (1 : 1 : 8, by volume) gas mixture, unless otherwise indicated.

### 16S rRNA gene phylogeny

The 16S rRNA gene of strain YZC6^T^ was amplified and sequenced using two universal primers, 27F and 1492R, as previously described [7]. The sequence of strain YZC6^T^ was used to identify closely related taxa by searching the Basic Local Alignment Search Tool (blast) (http://blast.ncbi.nlm.nih.gov/Blast.cgi) and the EzBioCloud databases [9]. The 16S rRNA gene sequences of closely related species and *Blautia hansenii* were obtained from the GenBank/EMBL/DDBJ database and aligned using muscle [10]. An evolutionary distance matrix was generated using Kimura’s two-parameter model [11]. A phylogenetic tree was reconstructed using the neighbour-joining method, as well as the maximum-likelihood and maximum-parsimony trees, using the mega software package version 11 [12]. Bootstrap analysis with 1000 replications was used to assess the reliability of the tree topologies [13]. Similarity values of the 16S rRNA gene sequences were calculated using EzBioCloud [9].

### Genome features

The genomic DNA of strain YZC6^T^, *L. amygdalina* DSM 12857^T^, and *L. xylanolytica* JCM 15735^T^ was prepared from cells harvested from the PYG medium using Qiagen genomic tips. Whole-genome paired-end sequencing of these strains was performed using an Illumina MiSeq system. After filtering low-quality reads and trimming adapter sequences [14], draft genomes were reconstructed using skesa (version 2.4.0) [15] and annotated using the DDBJ Fast Annotation and Submission Tool (version 1.6.0; https://dfast.ddbj.nig.ac.jp/) [16]. The completeness and contamination values of the genomes were calculated using CheckM [17]. Draft genomes of closely related species were obtained from the National Center for Biotechnology Information assembly database. A maximum-likelihood phylogenomic tree based on 335 conserved core genes was reconstructed using the PanACoTA pipeline (version 1.2.0) [18] and iq-tree (version 2.2.2.3) [19], using the best-fit model (GTR+F+I+R4) selected by ModelFinder [20]. The tree was visualized using iTOL version 6 [21]. The ANI values were calculated using IPGA version 1.09, which is available on the IPGA server (https://nmdc.cn/ipga/) [22]. The dDDH values were analysed using the Genome-to-Genome Distance Calculator 3.0, available on the DSMZ server (https://ggdc.dsmz.de/ggdc.php) [23]. The AAI values were computed using EzAAI 1.2.2 (http://leb.snu.ac.kr/ezaai) [24]. The circular genome map and cluster of orthologous groups (COG) of strain YZC6^T^ and the genus *Lacrimispora* were analysed using GenoVi [25]. The predicted protein sequences of strain YZC6^T^ were assigned Kyoto Encyclopedia of Genes and Genomes (kegg) Orthology identifiers (K numbers) using BlastKOALA (https://www.kegg.jp/blastkoala/) [26]. Metabolic pathways were reconstructed based on the assigned K numbers using kegg Mapper (https://www.kegg.jp/kegg/mapper/reconstruct.html) [27,28]. Secondary metabolite biosynthesis gene clusters (BGCs) were predicted using antiSMASH (version 7.0.1), with the detection strictness set to strict (https://antismash.secondarymetabolites.org) [29]. Bacterial pathogenicity was analysed using PathogenFinder (version 1.1; https://cge.food.dtu.dk/services/PathogenFinder/) [30]. Antimicrobial resistance genes were identified using ResFinder (version 4.5.0), with a threshold and minimum length of 90 and 60  %, respectively (https://cge.food.dtu.dk/services/ResFinder/) [31].

### Physiology and chemotaxonomy

Cell, spore, and colony morphology were observed after cultivation on RCM plates at 30  °C for 3 days under anaerobic conditions. Spore formation was examined after cultivation in PYG medium at 30  °C for 3 days under anaerobic conditions, after performing heat treatment at 75  °C for 15 min. Spore morphology was observed microscopically using the Schaeffer–Fulton-modified Wirtz staining method (Wirtz staining kit, Muto Pure Chemicals). Gram stain and catalase activity were determined as previously described [32].

The growth temperature, pH, and NaCl tolerance ranges of strain YZC6^T^ were determined in PYG medium under an H_2_/CO_2_/N_2_ (1 : 1 : 8) gas mixture for 7 days. The growth conditions of strain YZC6^T^ were tested at 10, 15, 20, 25, 37, 40, and 45 °C. The pH was adjusted to pH 4.5, 5.0, 5.5, 6.0, 6.7, 7.2, 7.8, 8.5, and 9.0 (as values verified after autoclaving) using 1 N HCl or 5 N NaOH. CHES (Good’s buffer; Dojindo Laboratories; 20 mM) was used to adjust the pH of the medium to >pH 8.5. The NaCl concentration ranged from 0 to 50 g l^−1^ NaCl (in 10 g l^−1^ intervals). Growth was assessed by measuring the optical density at 600 nm and the culture pH.

The isolate and reference-type strains were cultivated on RCM plates for 3 days under anaerobic conditions and characterized using API 20A and API ZYM (bioMérieux) test strips in duplicate, according to the manufacturer’s instructions. The fermentation products in the PYG medium were analysed after 3 days of culturing at 30  °C under an H_2_/CO_2_/N_2_ (1 : 1 : 8) gas mixture. Analysis of the respiratory quinones and polar lipids in the cell wall was conducted after cultivation in PYG medium at 30  °C for 24 h under an H_2_/CO_2_/N_2_ (1 : 1 : 8) gas mixture using the DSMZ identification service (Braunschweig, Germany). Organic acids present in the cultivated PYG medium were determined using high-performance liquid chromatography (Nexera series system, Shimadzu Corporation) with an ion exclusion column (Shim-Pak Fast OA, Shimadzu Corporation). The cellular fatty acids derived from cells cultured on RCM plates for 3 days were analysed using the ANAER6 database of the Microbial Identification System, as described previously [33].

## Results and discussion

### 16S rRNA gene phylogeny

**Fig. 1.**
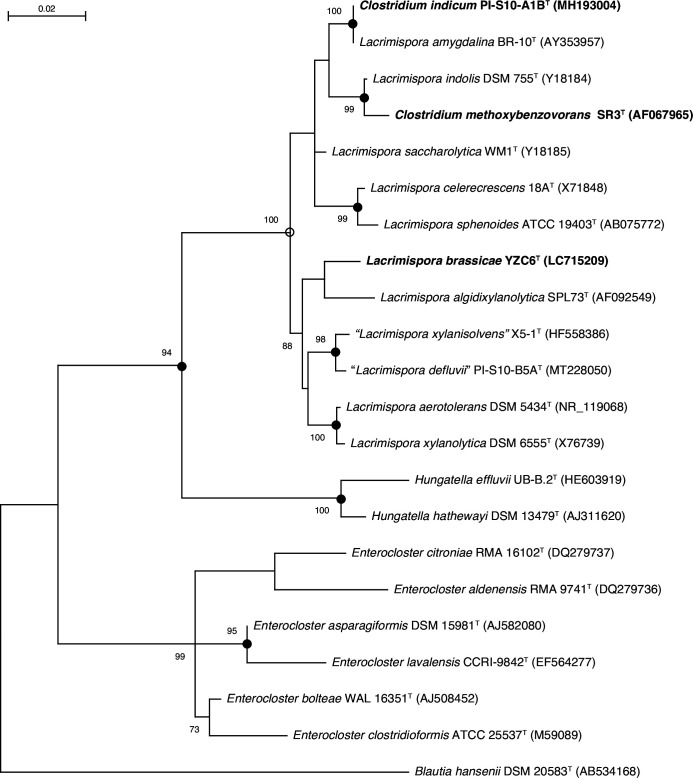
Maximum-likelihood tree based on 16S rRNA gene sequences, showing the phylogenetic relationships of strain YZC6^T^ with related taxa. The filled circles indicate the corresponding branches generated by maximum-likelihood (ML), neighbour-joining (NJ), and maximum-parsimony (MP) methods. Open circles indicate corresponding branches generated by the ML and NJ methods. Bootstrap percentages of >70  % (based on 1000 replications) are shown at the branch points. Bar: 0.02 substitutions per nucleotide position. The sequence of *Blautia hansenii* DSM 20583^T^ was used as an outgroup. Strain YZC6^T^ (proposed as a novel species in the present study), *Clostridium indicum* PI-S10-A1B^T^ (proposed as a later heterotypic synonym of *Lacrimispora amygdalina*), *Clostridium methoxybenzovorans* SR3^T^ (proposed as a later heterotypic synonym of *Lacrimispora indolis*) are in boldface type.

The length of the sequenced 16S rRNA gene was 1455 bp. Phylogenetic analysis based on the 16S rRNA gene sequences indicated that strain YZC6^T^ formed a distinct clade within the genus *Lacrimispora* (Figs 1, S1, and S2, available in the online version of this article). Strain YZC6^T^ was grouped with the type strains of *L. algidixylanolytica*, *L. aerotolerans*, *L. xylanolytica*, ‘*Lacrimispora xylanisolvens*’, and ‘*Lacrimispora defluvii*’ in the maximum-likelihood (Fig. 1), neighbour-joining (Fig. S1), and maximum-parsimony (Fig. S2) phylogenetic trees. The most closely related species to strain YZC6^T^ was *L. aerotolerans* DSM 5434^T^ (98.3  % sequence similarity), followed by *L. saccharolytica* WM1^T^ (98.1  %), *L. algidixylanolytica* SPL73^T^ (98.1  %), and *L. xylanolytica* ATCC 49623^T^ (98.0  %).

### Genome features

**Fig. 2.**
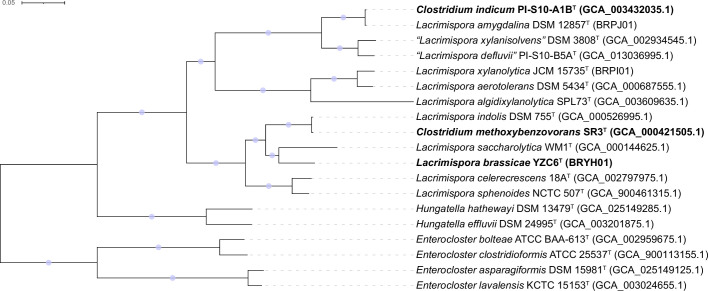
Phylogenetic relationship of strain YZC6^T^ and the type strains of the closely related taxa based on 335 core genes. The maximum-likelihood tree was reconstructed, and bootstrapping was set to 1000 replications. Light blue circles on the nodes mean bootstrap support of 100  %. Strain YZC6^T^ (proposed as a novel species in the present study), *Clostridium indicum* PI-S10-A1B^T^ (proposed as a later heterotypic synonym of *Lacrimispora amygdalina*), *Clostridium methoxybenzovorans* SR3^T^ (proposed as a later heterotypic synonym of *Lacrimispora indolis*) are in boldface type. The tree was rooted using midpoint rooting. Bar, 0.05 substitutions per nucleotide position.

The draft genome of YZC6^T^ comprised 6.06 Mbp, with a G+C content of 44.6  mol%, 5526 coding sequences, 64 tRNA genes, one rRNA gene, and one CRISPR (Table S1). The genome length of *Lacrimispora*-type strains ranged from 4.62 to 6.38 Mbp (Fig. S3), thereby situating YZC6^T^ as a relatively large genome within the genus. The G+C content of the whole-genome sequences of DSM 12857^T^ and JCM 15735^T^ were 42.3 and 41.9 mol%, respectively, with other genome sequence statistics provided in Table S1. A maximum-likelihood phylogenomic tree based on 335 conserved core genes is shown in Fig. 2. Strain YZC6^T^ was grouped within the same clade as *L. saccharolytica* WM1^T^. The ANI, dDDH, and AAI values between strain YZC6^T^ and the type strains of phylogenetically related species were 74.6–87.8 %, 20.0–36.1 %, and 77.4–90.4 %, respectively (Fig. S4, Table S2), which are below the 95, 70, and 95–96% thresholds proposed for species definition, respectively [34,37].

In 2019, Gundwar *et al.* proposed a novel endospore-forming strain PI-S10-A1B^T^ of the genus *Clostridium*, named *C. indicum*. The strain displayed high sequence similarity of the 16S rRNA gene to *L. amygdalina* BR-10^T^, and low rates of DNA–DNA relatedness were observed [38]. Recently, it was suggested that overall genomic relatedness indices, such as ANI and dDDH, should be used to identify new species [36,39]. However, genomic analyses between these closely related species have not yet been performed. In this study, we analysed the ANI, dDDH, and AAI values between strains *L. amygdalina* DSM 12857^T^ and *L. indicum* PI-S10-A1B^T^ and obtained values of 99.0, 95.8, and 98.6  %, respectively (Fig. S4, Table S2). These results indicate that the two strains belonged to the same species.

*C. methoxybenzovorans*, previously classified in the *Clostridium* XIVa cluster, was reclassified based on genome-based analysis, suggesting that it is more closely related to *L. indolis* [40]. However, *C. methoxybenzovorans* SR3^T^ is a valid species. The AAI value between *C. methoxybenzovorans* SR3^T^ and *L. indolis* DSM 755^T^ was found to be 99.2  %, exceeding the threshold for distinguishing prokaryotic species [37]. These results are consistent with previous findings on ANI and dDDH [41]; therefore, the two organisms should be considered the same species.

The 6197 protein-coding genes in the new isolate YZC6^T^ were clustered into 23 categories in the COG 2020 database using GenoVi. The most prevalent COG category in the genome of strain YZC6^T^ was carbohydrate transport and metabolism (G), comprising 15.1  % of the genome, followed by transcription (K) at 10.6%, signal transduction mechanisms (T) at 7.1%, and general function prediction only (R) at 7.1  %. Similar findings were obtained for the genus *Lacrimispora*, excluding * L. algidixylanolytica*, for which carbohydrate transport and metabolism (G) and transcription (K) were the most abundant COG categories (Figs S3, S5).

According to the PathogenFinder and ResFinder tools, no antibiotic resistance- or pathogenicity-related genes were detected in the genome of YZC6^T^. Secondary metabolites produced by bacteria are used as pharmaceutical raw material due to their antibacterial, antitumor, and antiviral activities [42,43]. According to the antiSMASH analysis, strain YZC6^T^ was found to encode eight secondary metabolite BGCs (Fig. S6). Seven of these, comprising five cyclic-lactone-autoinducer clusters and two ranthipeptide clusters, showed no significant similarity to known clusters. The remaining one was a non-ribosomal peptide synthetase (NRPS) cluster was similar to the ulbactin F/ulbactin G gene cluster (28  % of genes showed similarity with the MIBiG accession no. BGC0002472), suggesting the potential for their novelty. Members of the genus *Lacrimispora* harboured three to nine BGCs, cyclic-lactone-autoinducer and ranthipeptide gene clusters were commonly identified. Most of these gene clusters were unknown clusters. The cyclic-lactone-autoinducer cluster has possibility producing pro-peptides such as AgrD, which is associated with quorum sensing in Gram-stain-positive bacteria [44]. The putative ranthipeptides identified in *Clostridium beijerinckii* and *Clostridium ljungdahlii*, participate in quorum sensing and control of cellular metabolism [45]. Further studies are needed to elucidate whether the strain YZC6^T^ has the potential to produce novel secondary metabolites.

The family *Brassicaceae*, which include cabbages, is known for its high nitrate and nitrite content [46]. As strain YZC6^T^ was isolated from fermented cabbage, its genome was analysed with a focus on nitrogen metabolism, including nitrate and nitrite reduction. The results of the BlastKOALA analysis indicated that no nitrate reductase gene was found in the genome of strain YZC6^T^. Nevertheless, the genes encoding enzymes involved in N metabolism, such as nitrite reductase (EC 1.7.1.4), glutamate dehydrogenase (EC 1.4.1.4), glutamine synthetase (EC 6.3.1.2), glutamate synthase (NADPH; EC 1.4.1.13), and ferredoxin (EC 1.4.7.1), were identified (Fig. S7). Therefore, nitrite serves as the nitrogen source for strain YZC6^T^, offering insights into the strain’s ability to adapt to the fermented cabbage environment.

### Physiology and chemotaxonomy

The temperature range for growth of strain YZC6^T^ was 10–37 °C (optimal growth at 25 °C), with the strain growing at initial pH 5.5–8.5 (optimal growth at pH 7.8). The strain grew in a medium containing NaCl up to 20 g l^−1^. The final pH values of the grown cultures under these conditions ranged from pH 4.8 to 5.6. As shown in Table 1, several characteristics distinguished the strain YZC6^T^ from the type strains of closely related species, including *L. aerotolerans* JCM 15733^T^, *L. saccharolytica* DSM 2544^T^, *L. algidixylanolytica* DSM 12273^T^ and *L. xylanolytica* JCM 15735^T^. Strains YZC6^T^ and DSM 2544^T^ reacted positively to d-mannitol and produced indole, whereas the other type strains JCM 15733^T^, DSM 12273^T^, and JCM 15735^T^ did not. Strain YZC6^T^ reacted positively to cellobiose and melezitose, whereas strain DSM 2544^T^ did not. Furthermore, only strain YZC6^T^ displayed negative reactions for C8 esterase and α-galactosidase among the tested strains. Acetate and lactate were the principal fermentation products in the culture supernatant of YZC6^T^ grown in PYG medium. The predominant cellular fatty acids in strain YZC6^T^ were C_16 : 0_ and C_18 : 1_ *cis* 11 dimethyl acetal, which are the major components of closely related species. Notably, strain YZC6^T^ did not contain cellular fatty acids C_15 : 0_ anteiso 3OH /C_16 : 1_ *cis* 7 dimethyl acetal, C_16 : 0_ aldehyde, or C_14 : 0_ dimethyl acetal, which distinguished it from JCM 15733^T^, DSM 12273^T^, and JCM 15735^T^ (Table S3). Strain YZC6^T^ contained one unidentified glycoaminolipid, two unidentified lipids, four unidentified glycolipids, five unidentified phospholipids, and one identified phosphoglycolipid (Fig. S8). More detailed characteristics of the strain YZC6^T^ are provided below in the species description.

**Table 1.** Differential characteristics between *Lacrimispora brassicae* sp. nov. and their related species of the genus *Lacrimispora* Strains: 1, YZC6^T^; 2, *L. aerotolerans* JCM 15733^T^; 3, *L. saccharolytica* DSM 2544^T^; 4, *L. algidixylanolytica* DSM 12273^T^; 5, *L. xylanolytica* JCM 15735^T^. All tested characteristics were determined under identical conditions in the present study. +, Positive reaction; −, negative reaction.

**Characteristic**	1	2	3	4	5
Indole production	+	−	+	−	−
Acid production from:					
d-Mannitol	+	−	+	−	−
Cellobiose	+	+	−	+	+
Melezitose	+	+	−	+	+
d-Sorbitol	−	−	−	−	+
Trehalose	+	−	+	−	+
Enzyme production:					
Alkaline phosphatase	−	−	+	−	−
C8 esterase	−	+	+	+	+
α-Galactosidase	−	+	+	+	+
β-Glucosidase	+	+	−	+	+

## Conclusion

Based on the phylogenetic, phenotypic, and chemotaxonomic results, we propose that strain YZC6^T^ represents a novel species within the genus *Lacrimispora*, for which we propose the name *Lacrimispora brassicae* sp. nov. Furthermore, we propose to reclassify *C. indicum* Gundawar *et al*. 2019 as a later heterotypic synonym of *L. amygdalina* (Parshina *et al*. 2003) Haas and Blanchard 2020, and *C. methoxybenzovorans* Mechichi *et al*. 1999 as a later heterotypic synonym of *L. indolis* (McClung and McCoy 1957) Haas and Blanchard 2020.

## Description of *Lacrimispora brassicae* sp. nov.

*Lacrimispora brassicae* (bras′si.cae. L. gen. n. *brassicae*, of cabbage)

The cells are Gram-stain-negative, spore-forming, catalase-negative, and rod-shaped (0.5×1.5–3.3 µm). Colonies cultured for 48 h on RCM plates at 30  °C under anaerobic conditions are 0.5–1.2 mm in diameter, cream, convex, and circular. Growth occurs at 10–37  °C, at pH 5.5–8.5, and with NaCl concentrations lower than 2  % (w/v). Acetic and lactic acids are produced from PYG medium.

In the API 20A test (incubated at 30  °C for 48 h), the type strain shows a positive reaction for d-glucose, d-mannitol, lactose, sucrose maltose, salicin, d-xylose, l-arabinose, aesculin, cellobiose, d-mannose, melezitose, raffinose, l-rhamnose, and trehalose. Negative reactions for glycerol and d-sorbitol are observed. Indole is detected. Urease is not detected. Gelatin is not hydrolysed.

Using the commercial API ZYM test, the strain shows a positive reaction for C4 esterase, acid phosphatase, naphthol-AS-BI-phosphohydrolase, β-galactosidase, α-glucosidase, and β-glucosidase. It shows negative reactions for alkaline phosphatase, C8 esterase, C14 lipase, leucine aminopeptidase, valine aminopeptidase, cystine aminopeptidase, trypsin, chymotrypsin, α-galactosidase, β-glucuronidase, *N*-acetyl-β-glucosaminidase, α-mannosidase, and α-fucosidase activities.

The cells contain *meso*-diaminopimelic acid. No respiratory quinones are detected. The polar lipids include one unidentified glycoaminolipid, two unidentified lipids, four unidentified glycolipids, five unidentified phospholipids, and one identified phosphoglycolipid. The G+C content of the genomic DNA is 44.6 mol% (whole-genome analysis). The type strain is YZC6^T^ (=MAFF 212518^T^=JCM 32810^T^=DSM 112100^T^). The GenBank/EMBL/DDBJ accession number for the 16S rRNA gene sequence of strain YZC6^T^ is LC715209. The accession number for the draft genome sequence of strain YZC6^T^ is BRYH00000000.

## Emended description of *Lacrimispora amygdalina* (Parshina *et al.* 2003) Haas and Blanchard 2020

*Lacrimispora amygdalina* (a.myg.da.li′na. L. fem. adj. *amygdalina*, made from almonds, referring to the smell of benzaldehyde, which is produced by the type strain).

The description is given by Parshina *et al.* and Haas and Blanchard, with the following modifications: The originally reported G+C content was 32 mol%, but the whole-genome sequence shows 42.3 mol%. The accession number for the draft genome sequence of *Lacrimispora amygdalina* DSM 12857^T^ is BRPJ00000000.

The type strain is BR-10^T^ (=DSM 12857^T^=ATCC BAA-501^T^). *Clostridium indicum* Gundawar *et al*. 2019 is considered a later heterotypic synonym of *Lacrimispora amygdalina* (Parshina *et al*. 2003) Haas and Blanchard 2020.

## supplementary material

10.1099/ijsem.0.006456 Uncited Supplementary Material 1.

## Abbreviations

AAI: average amino acid identity
ANI: average nucleotide identity
BGC: biosynthesis gene cluster
COG: cluster of orthologous groups
dDDH: digital DNA–DNA hybridization
PYG: peptone–yeast extract–glucose
RCM: reinforced clostridial medium
