# Supplementary material for: Lacrimispora brassicae sp. nov. isolated from fermented cabbage, and proposal of Clostridium indicum Gundawar et al. 2019 and Clostridium methoxybenzovorans Mechichi et al. 1999 as heterotypic synonyms of Lacrimispora amygdalina (Parshina et al. 2003) Haas and Blanchard 2020 and Lacrimispora indolis (McClung and McCoy 1957) Haas and Blanchard 2020, respectively
Source: Int J Syst Evol Microbiol. 2024 Jul 17;74(7):006456. doi: 10.1099/ijsem.0.006456 (PMC11316579; doi:10.1099/ijsem.0.006456)
Supplement: Uncited Supplementary Material 1. [file ijsem-74-06456-s001.pdf]

IJSEM supplementary materials for:

***Lacrimispora brassicae* sp. nov. isolated from fermented cabbage, and proposal of *Clostridium indicum* Gundawar *et al*. 2019 and *Clostridium methoxybenzovorans* Mechichi *et al*. 1999 as heterotypic synonyms of *Lacrimispora amygdalina* (Parshina *et al*. 2003) Haas and Blanchard 2020 and *Lacrimispora indolis* (McClung and McCoy 1957) Haas and Blanchard 2020, respectively**

Author names

Hisami Kobayashi[1], Yasuhiro Tanizawa[2], Mitsuo Sakamoto[3], Moriya Ohkuma[3], and Masanori Tohno[1,4,5]

Affiliation

[1]Institute of Livestock and Grassland Science, National Agriculture and Food Research Organization, Nasushiobara, Tochigi 329-2793, Japan; [2]Department of Informatics, National Institute of Genetics, Mishima, Shizuoka 411-8540, Japan; [3]Microbe Division/Japan Collection of Microorganisms, RIKEN BioResource Research Center, Tsukuba, Ibaraki 305-0074, Japan; [4]Research Center of Genetic Resources, National Agriculture and Food Research Organization, Tsukuba, Ibaraki 305-8602, Japan; [5]University of Tsukuba, Graduate School of Science and Technology, 305-8602 Tsukuba, Japan.

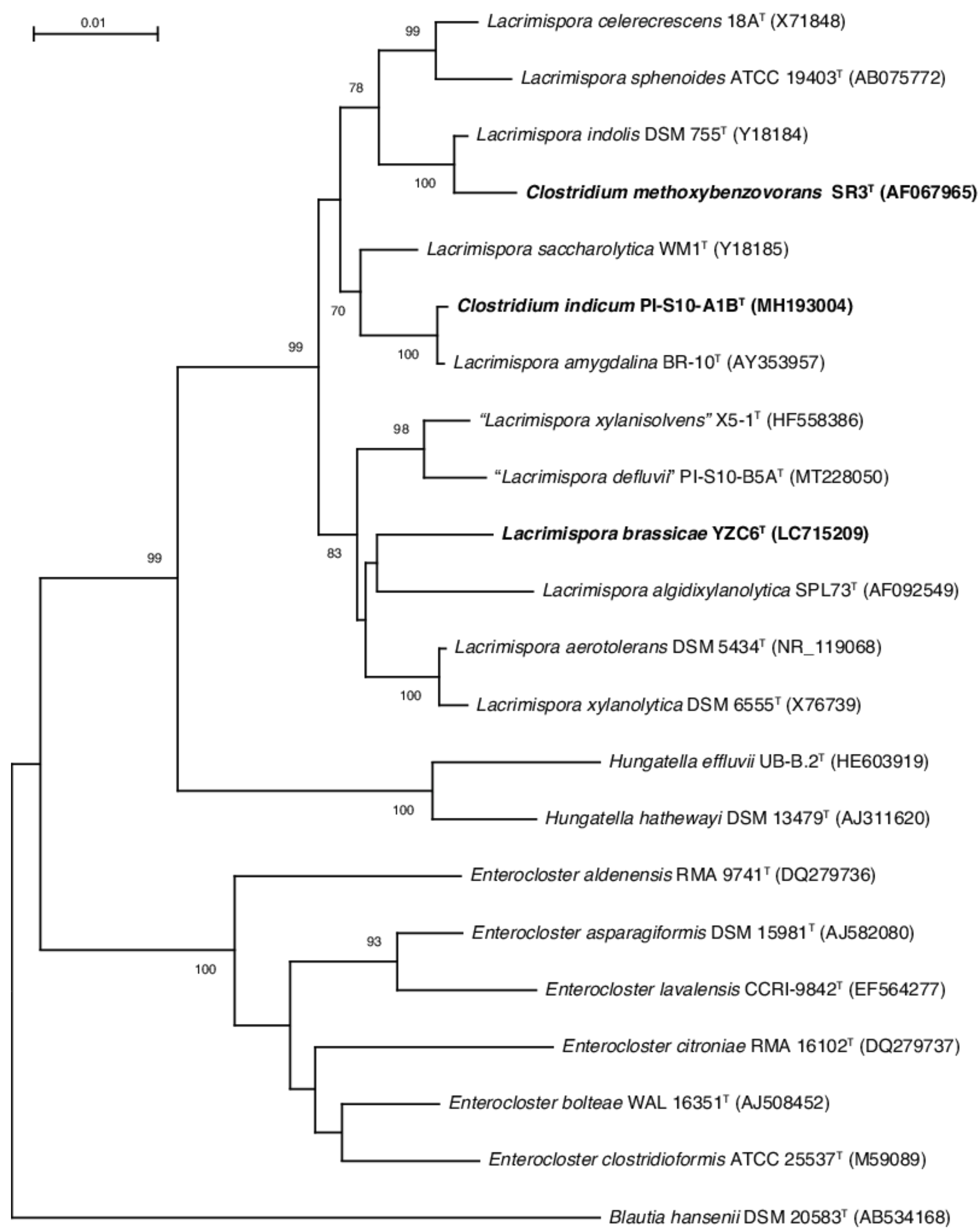

Supplementary Fig. S1. The neighbor-joining tree based on 16S rRNA gene sequences, showing the phylogenetic relationship of strain YZC6^T within members of the genus *Lacrimispora* and neighboring genera. The sequence of *Blautia hansenii* DSM 20583^T was used as an outgroup. Bootstrap percentages greater than 70% (based on 1000 replications) are shown at branch points. Bar: 0.01 substitutions per nucleotide position. Strain YZC6^T (proposed as a novel species in the present study), *Clostridium indicum* PI-S10-A1B^T (proposed as a later heterotypic synonym of *Lacrimispora amygdalina*) and *Clostridium methoxybenzovorans* SR3^T (proposed as a later heterotypic synonym of *Lacrimispora indolis*) are in boldface type.

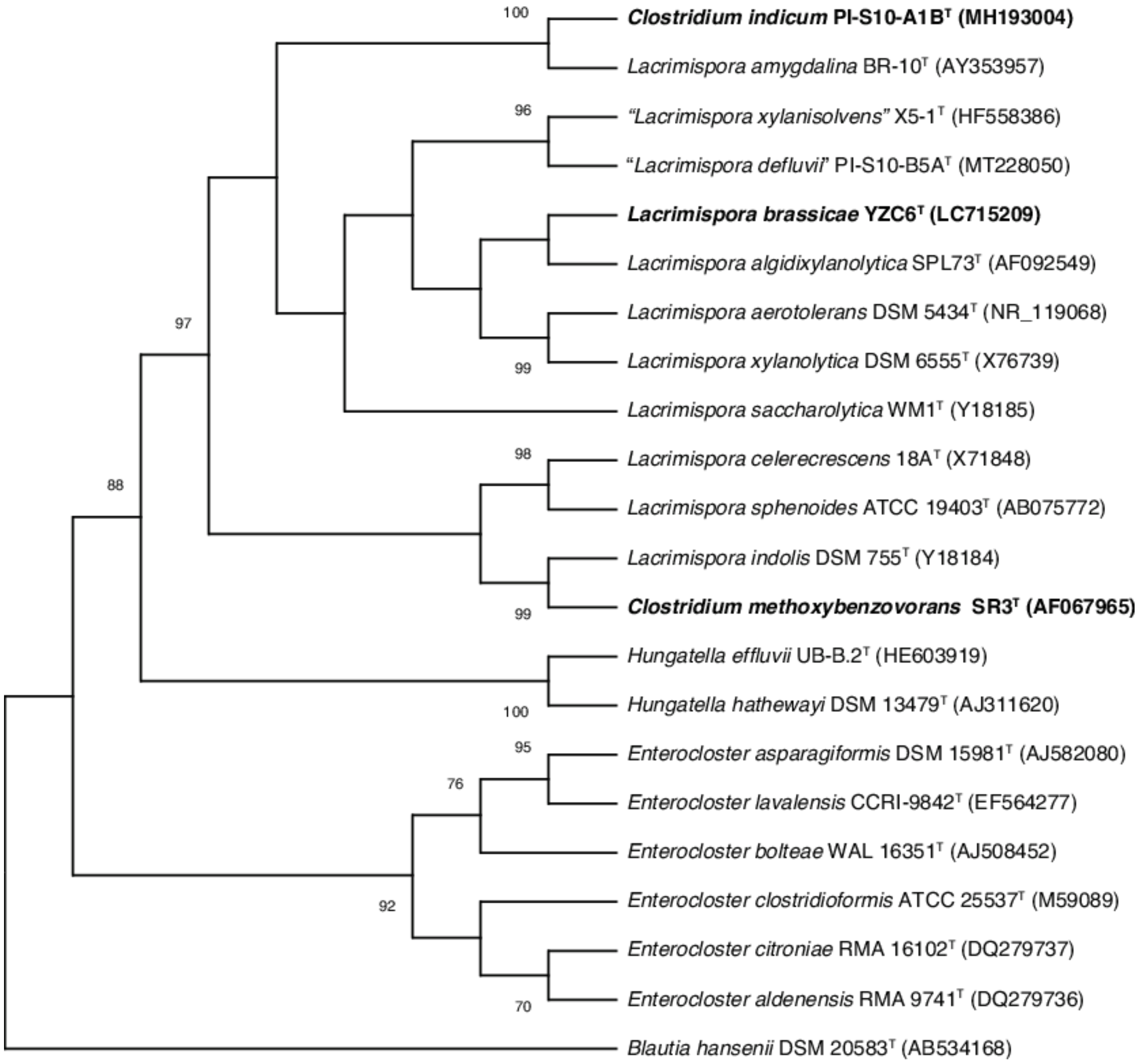

Supplementary Fig. S2. The maximum-parsimony tree based on 16S rRNA gene sequences, showing the phylogenetic relationship of strain YZC6^T within members of the genus *Lacrimispora* and neighboring genera. The sequence of *Blautia hansenii* DSM 20583^T was used as an outgroup. Bootstrap percentages greater than 70% (based on 1000 replications) are shown at branch points. Strain YZC6^T (proposed as a novel species in the present study), *Clostridium indicum* PI-S10-A1B^T (proposed as a later heterotypic synonym of *Lacrimispora amygdalina*) and *Clostridium methoxybenzovorans* SR3^T (proposed as a later heterotypic synonym of *Lacrimispora indolis*) are in boldface type.

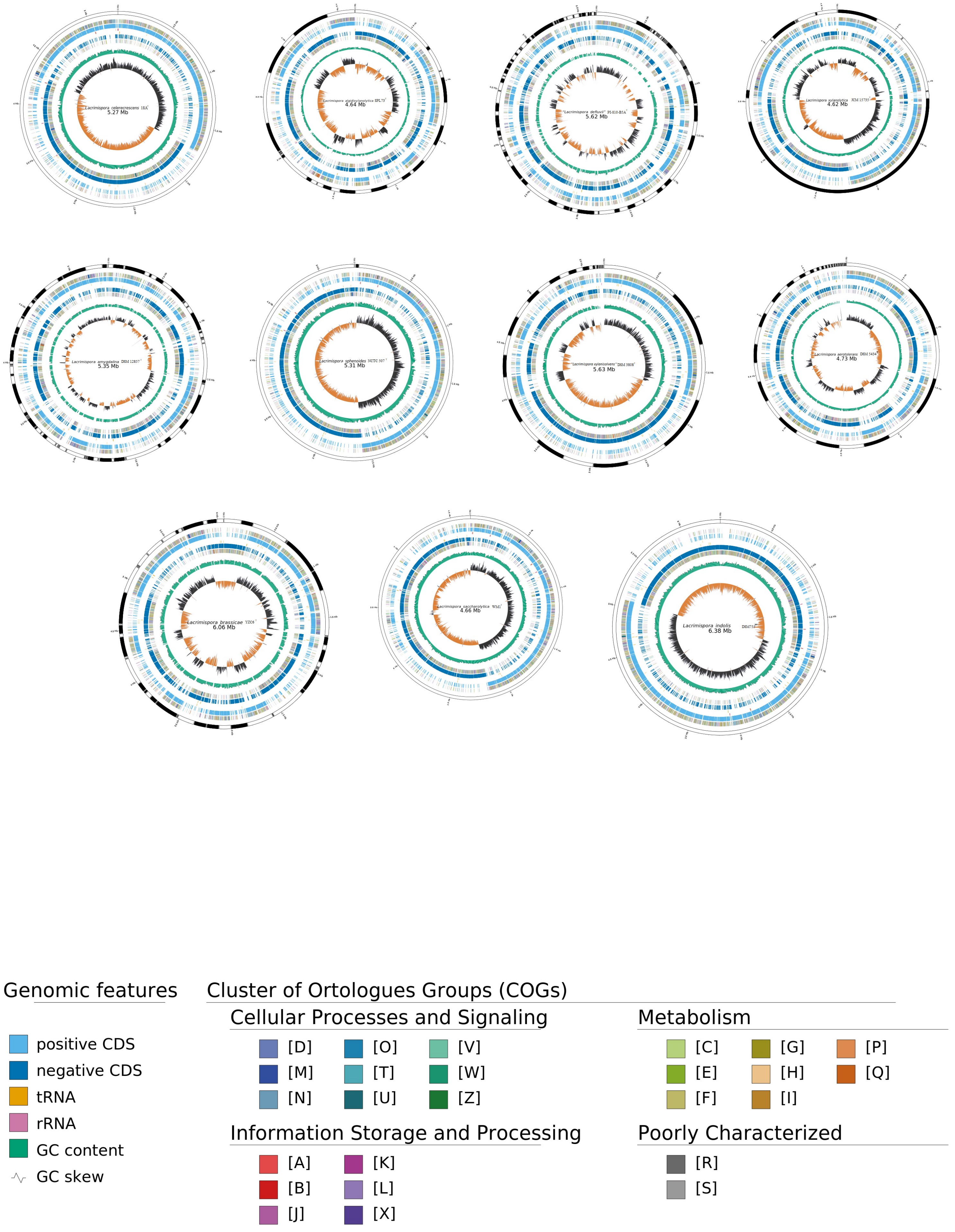

Supplementary Fig. S3. Circular genomic representation of *Lacrimispora* strains

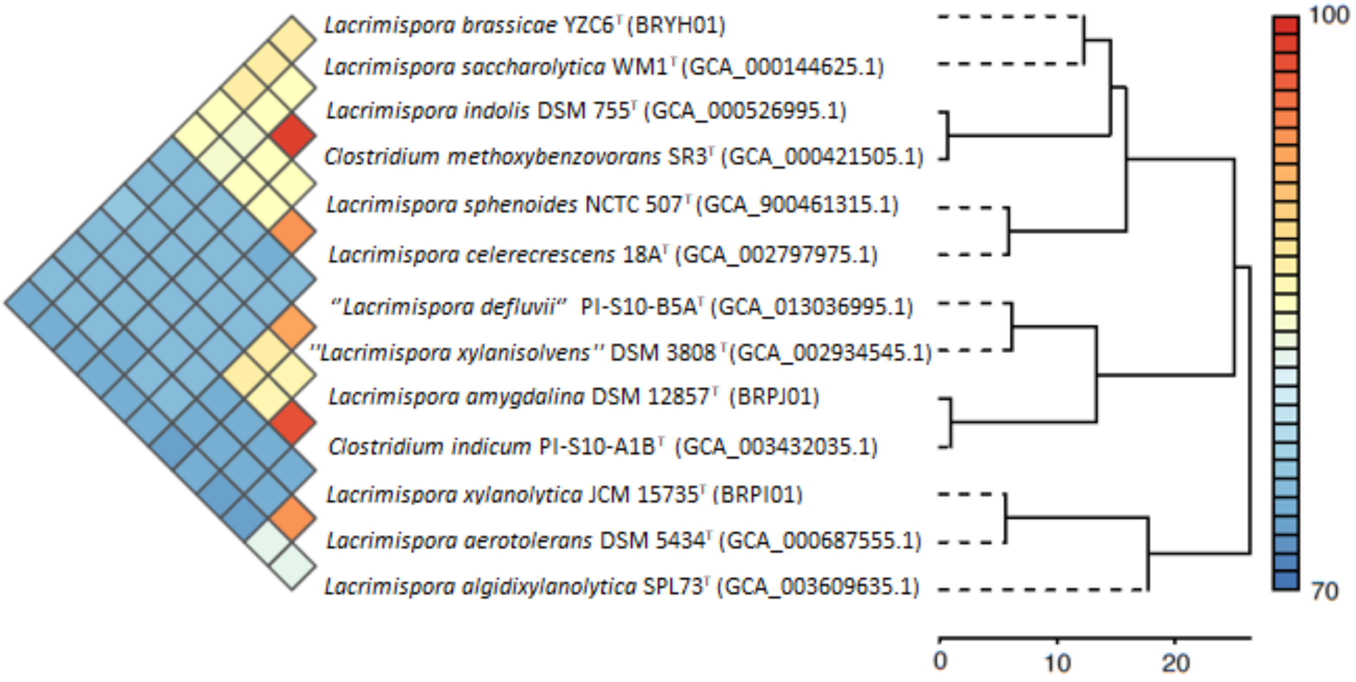

Supplementary Fig. S4. Pairwise comparisons of average nucleotide identity (ANI) values between strain YZC6^T and related taxa. The color indicates the value of ANI, the value range is 70-100 with color turning from blue to red. The genome distance is (1-ANI)%.

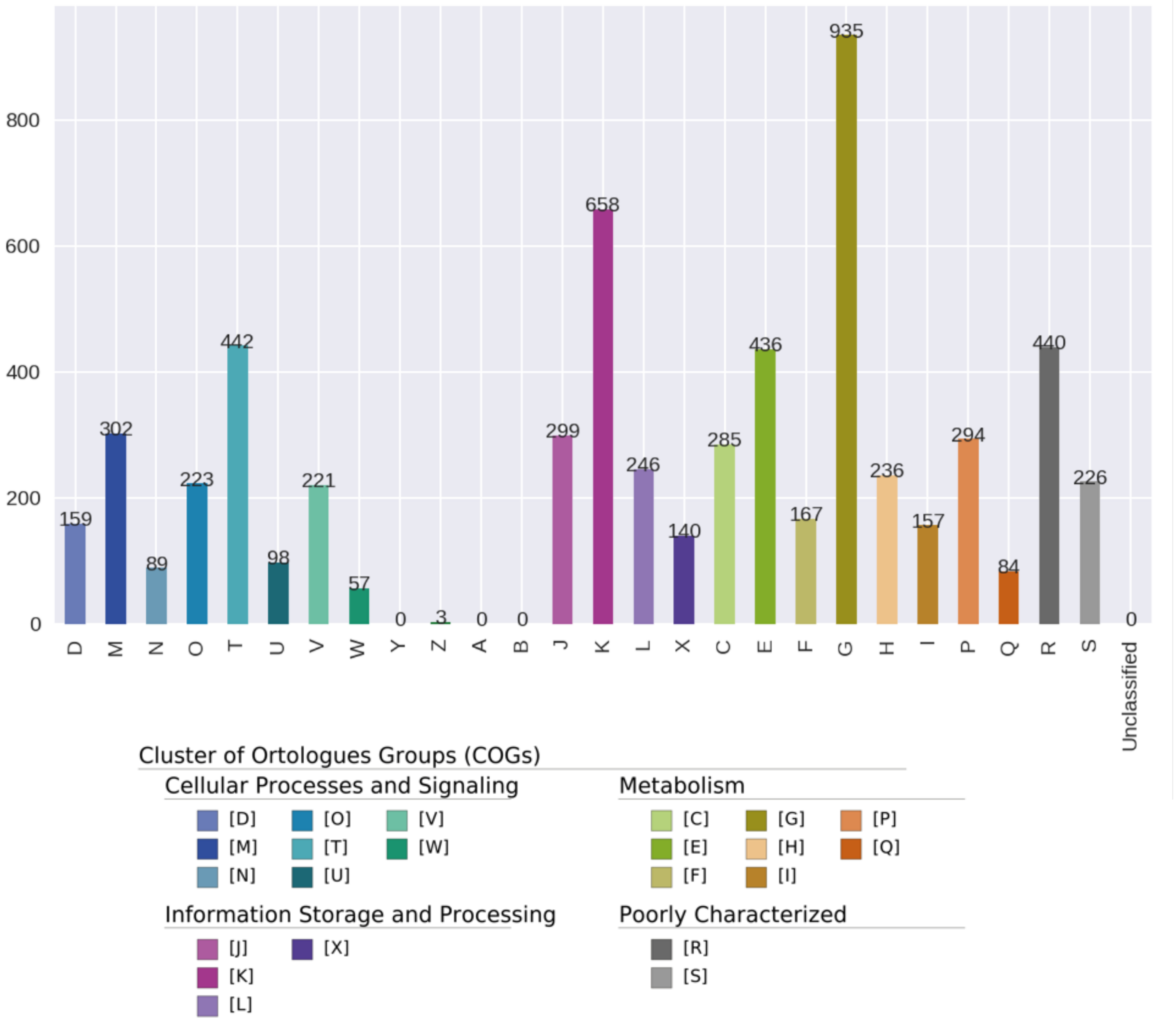

Supplementary Fig. S5. Proportions of genes associated with each clusters of orthologous group (COG) category of *Lacrimispora brassica* YZC6^{T}. D, D - Cell cycle control, cell division, chromosome partitioning; M, Cell wall/membrane/envelope biogenesis; N, Cell motility; O, Posttranslational modification, protein turnover, chaperones; T, Signal transduction mechanisms; U, Intracellular trafficking, secretion, and vesicular transport; V, Defense mechanisms; W, Extracellular structures; Y, Nuclear structure; Z, Cytoskeleton; A, RNA processing and modification; B, Chromatin structure and dynamics; J, Translation, ribosomal structure and biogenesis; K, Transcription; L, Replication, recombination and repair; X, Mobilome: prophages, transposons; C, Energy production and conversion; E, Amino acid transport and metabolism; F, Nucleotide transport and metabolism; G, Carbohydrate transport and metabolism; H, Coenzyme transport and metabolism; I, Lipid transport and metabolism; P, Inorganic ion transport and metabolism; Q, Secondary metabolites biosynthesis, transport and catabolism; R, General function prediction only; S, Function unknown;

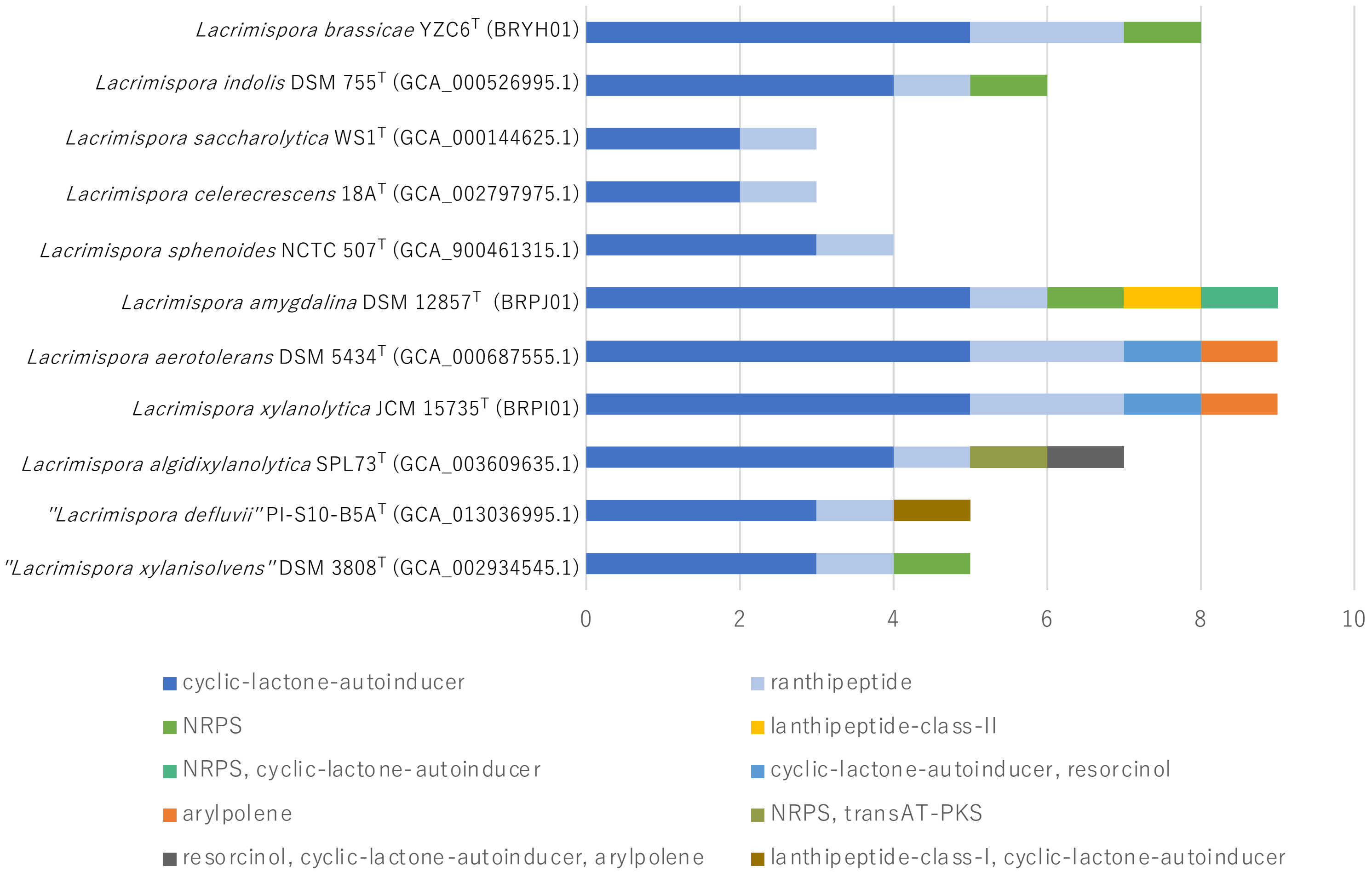

Supplementary Fig. S6. Number of predicted BGCs in the genomes of strain YZC6^T and closely related species in the genus *Lacrimispora* using antiSMASH 7.0.

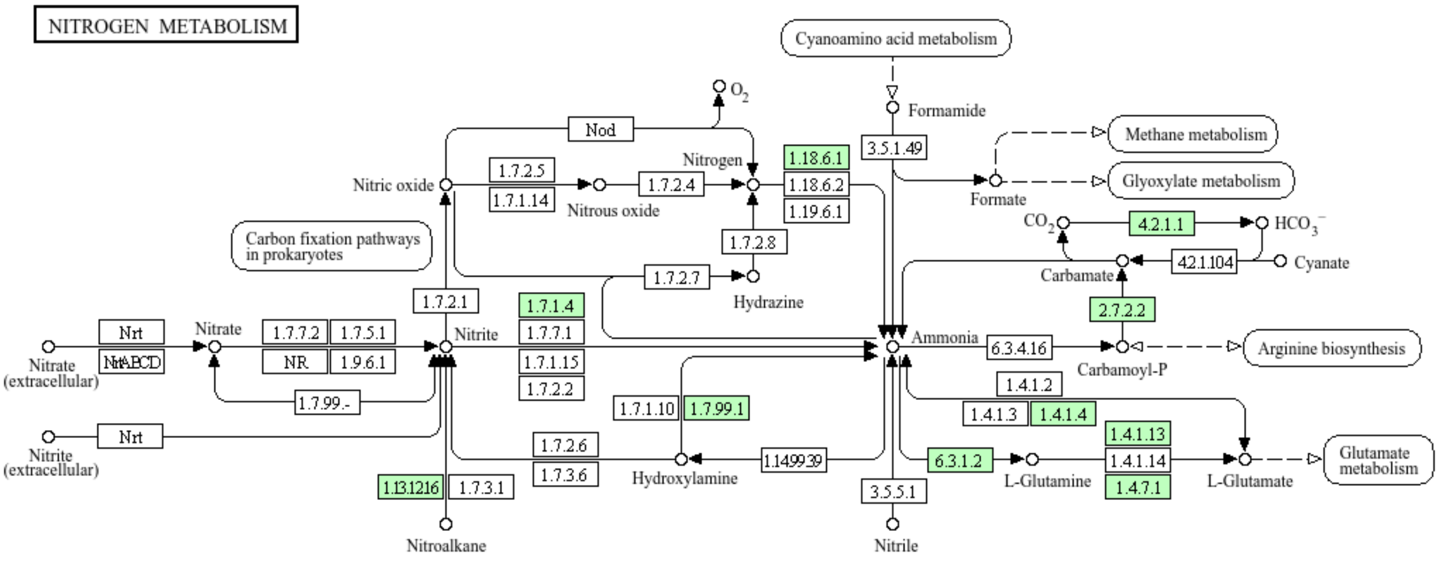

Supplementary Fig. S7. Nitrogen metabolic pathways of YZC6^T reconstructed using KEGG mapper. The numbers in the box indicate Enzyme Commission numbers. Green boxes indicated the presence of the specific genes in the genome of YZC6^T.

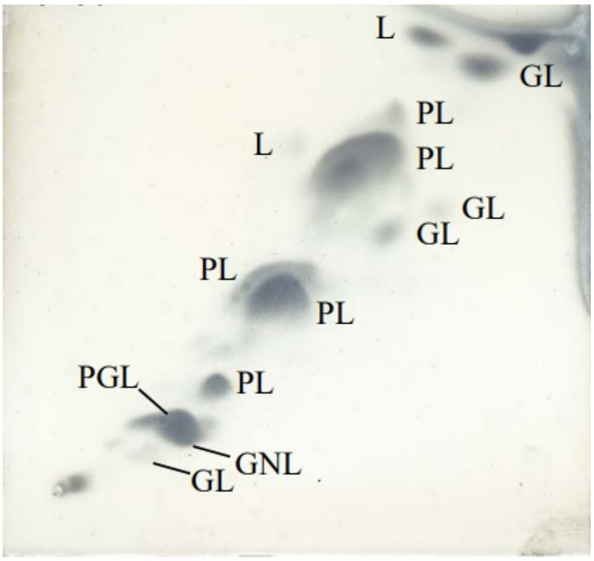

Supplementary Fig. S8. Polar lipid profile of strain YZC6^T. L, lipid; GL, glycolipid; PL, phospholipid; GNL, glycoaminolipid; PGL, phosphoglycolipid.

Supplementary Table S1. Summary of whole-genome sequencing in this study.

	YZC6^T	DSM 12857^T	JCM 15735^T
Accession number	BRYH01	BRPJ01	BRPI01
Number of sequences	65	103	21
Total length (bp)	6,059,566	5,350,244	4,620,800
N50 (bp)	216,902	108,220	484,068
G+C ratio (%)	44.6	42.3	41.9
Gap (%)	0.000	0.000	0.000
Depth coverage	191	217	237
Completeness (%)	99.38	99.38	98.75
Contamination (%)	2.37	3.74	2.28

Supplementary Table S2. The digital DNA-DNA hybridization (dDDH) values and average amino acid identity (AAI) between strain YZC6^{T} and other closely related species.

Strain No.	Strain	AAI (%)													DDBJ/ENA/GenBank accession number
		1	2	3	4	5	6	7	8	9	10	11	12	13	
1	*Lacrimispora brassicae* YZC6^{T}	-	90.4	90.3	88.7	88.5	78.5	78.2	77.9	77.9	77.9	77.4	90.3	77.7	BRYH01
2	*Lacrimispora indolis* DSM755^{T}	35.9	-	89.8	88.5	88.5	78.2	78.1	78.1	77.9	77.8	77.6	**99.2**	77.9	GCA_000526995.1
3	*Lacrimispora saccharolytica* WM1^{T}	35.7	31.8	-	88.0	88.0	78.3	77.7	78.1	78.2	78.1	77.4	89.0	78.2	GCA_000144625.1
4	*Lacrimispora celerecrescens* 18A^{T}	31.4	30.7	28.7	-	95.7	78.3	78.2	78.3	78.1	78.1	77.7	88.5	78.2	GCA_002797975.1
5	*Lacrimispora sphenoides* NCTC 507^{T}	31.4	30.5	28.6	56.0	-	78.1	77.9	78.0	78.1	77.9	77.4	88.5	77.9	GCA_900461315.1
6	*Lacrimispora amygdalina* DSM 12857^{T}	21.5	20.8	21.4	20.3	20.5	-	90.7	77.2	77.3	90.2	76.7	78.0	**98.6**	BRPJ01
7	‘’*Lacrimispora defluvii*‘’ PI-S10-B5A^{T}	21.4	20.7	20.8	20.6	20.2	33.7	-	76.9	77.0	95.7	76.2	77.6	90.8	GCA_013036995.1
8	*Lacrimispora aerotolerans* DSM 5434^{T}	20.5	20.1	19.9	20.2	20.2	19.4	19.7	-	96.4	77.5	87.3	78.0	77.0	GCA_000687555.1
9	*Lacrimispora xylanolytica* JCM 15735^{T}	20.5	20.3	20.1	20.5	20.3	19.3	19.4	57.6	-	77.0	87.2	77.8	77.3	BRPI01
10	‘’*Lacrimispora xylanisolvens*‘’ DSM 3808^{T}	20.5	20.7	20.7	20.7	20.4	32.5	57.4	20.2	19.5	-	76.7	77.6	90.2	GCA_002934545.1
11	*Lacrimispora algidixylanolytica* SPL37^{T}	20.0	20.0	20.2	20.0	19.9	19.1	18.8	25.8	25.5	19.5	-	77.4	76.6	GCA_003609635.1
12	*Clostridium methoxybenzovorans* SR3^{T}	36.1	**94.5**	31.7	30.7	30.4	20.8	20.8	20.2	20.3	20.6	20.1	-	77.6	GCA_000421505.1
13	*Clostridium indicum* PI-S10-A1B^{T}	20.9	20.9	21.5	20.6	20.7	**95.8**	34.1	19.4	19.6	32.6	19.1	21.1	-	GCA_003432035.1
		dDDH* (%)													

*Distances are inferred using Formula 2 (identities/high scoring segment pair length).

Table S3 Cellular fatty acid compositions of *Lacrimispora brassicae* sp. nov. and their related species of the genus *Lacrimispora*

Taxa: 1, YZC6^T; 2, *L. aerotolerans* JCM 15733^T; 3, *L. algidixylanolytica* DSM 12273^T; 4, *L. xylanolytica* JCM 15735^T. Each value shown is expressed as a percentage of the total fatty acids. The major fatty acids of each species are highlighted in bold. All tested fatty acid profiles were determined under identical conditions in the present study. -, not detected; DMA, dimethyl acetal; ALDE, aldehyde.

**Fatty acid**	**1**	**2**	**3**	**4**
Saturated:				
$C_{14:0}$	4.4	5.6	4.5	5.7
$C_{14:0}$ DMA	-	-	-	1.1
$C_{16:0}$ ALDE	-	-	1.4	1.2
$C_{16:0}$	**31.1**	**37.7**	**31.7**	**39.3**
$C_{16:0}$ DMA	1.8	3.7	8.6	6.8
Unsaturated:				
$C_{16:1}$ *cis* 7	1.8	2.2	1.9	1.2
$C_{16:1}$ *cis* 9	7.5	4.4	5.5	3.3
$C_{16:1}$ *cis* 9 DMA	6.8	**14.5**	7.1	**10.2**
$C_{18:1}$ *cis* 9	2.1	-	3.3	1.1
$C_{18:1}$ *cis* 9 DMA	8.8	5.2	6.5	5.6
$C_{18:1}$ *cis* 11 DMA	**22.2**	**13.6**	**14.6**	**14.1**
Summed features*:				
4	1.8	4.5	2.1	2.7
6	-	2.1	-	1.6
7	1.3	-	-	0.9
8	3.6	2.2	2.3	2.4
10	6.8	4.3	**10.5**	2.9

* Summed features are fatty acids that cannot be resolved reliably from another fatty acid using the chromatographic conditions chosen. The MIDI system groups these fatty acids together as one feature with a single percentage of the total. Summed feature 4: unknown 14.762/$C_{15:2}$ fatty acid/$C_{15:2}$/$C_{15:1}$ *cis* 7; summed feature 6: $C_{15:0}$ *anteiso* 3OH/$C_{16:1}$ *cis* 7 DMA; summed feature 7: $C_{17:2}$/$C_{17:1}$ *cis* 8 ; summed feature 8: $C_{17:1}$ *cis* 9/$C_{17:2}$; summed feature 10: $C_{18:1}$ *c*11/*t*9/*t*6/unknown 17.834.
